# Coping Strategies Used by Iranian Nurses to Deal With Burnout: A Qualitative Research

**DOI:** 10.5539/gjhs.v6n6p273

**Published:** 2014-08-15

**Authors:** Mohammad Mehdi Salaree, Armin Zareiyan, Abbas Ebadi, Mohammad Salaree

**Affiliations:** 1 School of Nursing, Baqiyatallah University of Medical Sciences, Tehran, IR Iran; 2 School of Nursing, AJA University of Medical Sciences. Tehran, IR Iran; 3 School of Health, Environmental Health Engineering Department, Shahid Sadoughi Univercity of Medical Sciences, IR Iran

**Keywords:** religion, spirituality, coping strategy, burnout professional, nurses, qualitative research, Iran

## Abstract

**Background:**

Although numerous studies have reported about coping strategies among health care worker throughout the world, but no research-based data are available on the perception of coping strategy among Clinical nurses in the Islamic Republic of Iran.

**Objective:**

The aim of the present study was to describe and explore the experiences of Iranian nurses about their coping strategies.

**Methods:**

In this study we used a qualitative research approach to explore how Iranian nurses perceive and resolve their burnout at work. Twelve nurses were selected by purposive sampling and in-depth semi structured interviews were conducted. All interviews were tape recorded, transcribed verbatim and then analyzed by means of the conventional qualitative content analysis method.

**Results:**

The 5 main themes that evolved from content analysis included “religious responsibility”, “approximation to God”, “spiritual reward”, “Holiness of the job” and “spiritual journey” emerged as the most important among these.

**Conclusions:**

The results of this study emphasized that religious or spiritual beliefs give purpose and meaning to nursing interventions, help them tolerate the problems at work, and make nursing care pleasurable. Therefore, although burnout is an important issue in nursing, attending to this dimension of their job is essential and healthcare authorities should pay a special attention to it.

## 1. Introduction

The quality of health care depends on many factors, including the health and work ability of health-care workers (Knezevic et al., 2011).There is growing understanding that work-related stress negatively affects the health of workers (Abdi, Kaviani, & Momeni, 2007; Raeissi & Tavakoli, 2003). As Chang et al. (2006) stated work stressors can be defined as ‘antecedent conditions within one’s job or the organization which require adaptive responses on the part of the employees’.

Numerous studies in different parts of the world (Golubic, 2009; Rahmani, 2010; Khaghanizadeh, 2008; Zandi, 2011; Garrosa, 2010) have shown that occupational stress is prevalent among nurses (Garrosa, Rainho, Moreno-Jiménez, & Monteiro, 2010; Golubic, Milosevic, Knezevic, & Mustajbegovic, 2009; Jennings, 2008; Kahghanizadeh, Sirati, & Abdi, 2008; Rahmani, Behshid, & Zamanzadeh, 2010; Zandi, Sayari, Ebadi, & Sanainasab, 2011). Certain work-related stressors involved in nursing, including exposure to pain, death and dying, role stress, lack of support of supervisors, misguided expectations, inadequate physical conditions (i.e., noise pollution), interpersonal conflicts, communication problems, lack of knowledge and insufficient social support, (Beckstead, 2002; Kalemoglu & Keskin, 2006; Schmitz, Neumann, & Oppermann, 2000), caring for extremely large numbers of patients, heavy work load disturbances in sleep pattern, shift work and long working hours, have been demonstrated to be major stressors (Kalemoglu & Keskin, 2006; Schreuder et al., 2011; Tekindal, Tekindal, Pinar, Ozturk, & Alan, 2012).

Fredeunberger (1974) stated that burnout occurred more commonly in occupations whose members directly work with people (Demir, Ulusoy, & Ulusoy, 2003). In this regard Adali and Priami (2002) considered burnout as a type of professional stress and also defined burnout as a prolonged response or “psychological syndrome” in response to chronic interpersonal stressors on the job (Ben-Zur & Michael, 2007; Lynch & Research, 2007; Martinussen, Richardsen, & Burke, 2007). Research findings, related to burnout indicated that burnout has negative consequences for the individual as well as for the organization at large. For example, relationships have been found between burnout and depression, a sense of failure, fatigue, and loss of motivation (Ahola & Hakanen, 2007; Euwema, Kop, & Bakker, 2004), medication use, and thoughts of suicide, job dissatisfaction and desire to leave the job, as well as number of sick days, absenteeism from work, and abandoning nursing. Finally, nursing burnout results in poor patient care, job dissatisfaction, lack of marital and familial harmony, decrease in self-esteem, difficulty in concentrating, social isolation, fatigue, loss of libido, headache, cold, gastrointestinal problems, sleep disorders, and alcohol and drug abuse (Demir et al., 2003; Vela-Bueno et al., 2008).

Individuals who are burnout feel psychologically drained or emotionally exhausted. They feel that their coping resources are being severely taxed by their work, and they feel “at the end of their rope.” (Wilcox, 2000). Thus, whenever coping resources that are inadequate and ineffectual can strongly dispose a worker to burnout (Myendeki, 2008). The use of coping resources can act as a buffer that interacts with a stressor to predict mental and behavioral outcomes (Santos, Barros, & Carolino, 2010). Studies have explored the coping strategies used by nurses to handle their stress (Wong, Leung, & So, 2001). Wang et al. (2011) found that the coping strategies used by nurses may vary with respect to their personal, psychological, and cultural factors (Wang, Kong, & Chair, 2011).

Spiritual values are increasingly recognized as necessary aspects of clinical care (Culliford, 2002), and contributes to health in many persons (Yuen, 2008).Today, many studies demonstrating health benefits of spiritual are many and growing in number. In relation to the impacts of coping strategies for the mental health of nurses, and Draper (2001), Wong et al. (2001), Koenig (2008) and Ravari et al. (1390) found that spiritual coping strategies may help the individual to find meaning and purpose in illness, resulting in self-empowerment to cope with the current stress until adaptation takes place. Despite the initial use of the term “burnout” in 1974 and decades of research, only a few studies have examined burnout and process of coping strategy in nurses especially in IR Iran. The purpose of this study was to obtain a deeper understanding and exploring of how nurses’ experiences and coping with burnout in IR Iran so that preventive interventions can be developed.

## 2. Method

A qualitative research method was used to explore burnout among Iranian nurses and the coping strategies adopted by them to deal with burnout. The aim of a qualitative study is to describe the lived experiences of the people who participated in the research (Flick, von Kardorff, & Steinke, 2004). In this study, participants were nurses, who had experienced long-term stress or high levels of burnout from their work and working in the various wards hospitals. The 21 clinical nurses selected by purposeful sampling and interviewed by the first researcher with the aim of capturing their experiences in the coping strategy on the burnout. The inclusion criterion for their was having a minimum of five years of work experience. After obtaining their informed consent, nurses were given an appointment according to their preferred date and time. The place of the interview was according to the participant’s preference; in a private place in the ward or their office. Each interview began with an open and broad question, such as: “Would you like to tell me about your experience of having burnout and how did you control it?”, or, “Tell me about how are you coping with burnout in your job”. The interviews lasted between 55 and 90 minutes. Interviews were tape-recorded and transcribed verbatim.

Data collection and analysis were performed simultaneously. Content analysis was based on scrutiny of the transcripts. According to Zhang and Wildemuth (2009) Content analysis is a research method for making replicable and valid inferences from data to their context, with the purpose of providing knowledge, new insights, a representation of facts and a practical guide to action. Analyses of textual data were with the conventional approach of interviews about the experience. In conventional content analysis, coding categories are derived directly from the text data. With a directed approach, analysis starts with a theory or relevant research findings as guidance for initial codes (Hsieh & Shannon, 2005).

### 2.1 Data Trustworthiness

Providing credibility checks are important for the trustworthiness of the findings (Gustafsson, Hassmén, Kenttä, & Johansson, 2008). Member checks were performed during the data analysis to assure the correct data interpretation and increasing the credibility of the findings. The researchers made every effort to clarify participants’ perceptions and the emergent themes to determine whether the codes and themes identified were appropriate to their experiences. The participants were contacted for verification of analyzing data from the full interview transcript and the summary. Maintaining long-term communication with the participants helped the researcher to establish trust and reach a better understanding of participants’ in the field. Two faculty members served as peer reviewers to ensure that no data were lost in the process of analysis.

## 3. Results

In-depth interviews were conducted with 21 Clinical nurses. The age ranged from 30 to 53 years, (mean age of 42.7). Twelve participants were female and nine were male. Eleven had a bachelor’s degree and ten had master’s degrees. All the participants worked in various wards such as medical surgical, orthopedics, intensive care, and the emergency department at Tehran hospitals in IR Iran (See Table 1).

**Table 1:** Demographic characteristics of the Participants

Demographic characteristics	Number	%
**Sex**		
Female	12	57/2
Male	9	42/8
**Marital Status**		
Married	16	76/2
Single	5	24/8
**Education Level**		
MSc	11	52/3
BSc	10	47/7
**Occupation Status**		
Head nurse	4	19
Staff nurse	14	66/7
Instructor	3	14/3

The participants in this study considered religious beliefs as one of the influential factors for adjustment with professional nursing tensions. They believed that spirituality gives purpose and meaning to nursing interventions. What makes them stay and continue their work despite all the occupational pressure and personal problems is the spiritual outlook they possess and that they do their task seeking closeness to God. Qualitative content analysis of the data (derived from the interviews) led to 5 themes: religious responsibility, approximation to God, spiritual reward, Holiness of the job, and spiritual journey. The following represents some of the participants’ comments regarding religious beliefs and the effect of spirituality on adjustment process and occupational burnout.

### 3.1 Religious Duty

A 46-year-old male nurse with war experience and over 2 decades of nursing experience says:

“… Even after 20 years of working… when I enter the hospital, it’s like entering a sacred place. I begin my work in the name of God…. I consider this job a religious duty for my brothers and fellow humans. I believe they are my brothers.”

In this regard, a male nurse with 21 years of clinical experience said: “Yes, … I consider helping a man in need not just a duty but part of my religious training. So when I work for the sake of God, I enjoy it. Why? Because I believe caring for a patient is a divine duty.”

### 3.2 Seeking Closeness to God

Many participating nurses had an indescribable feeling about their job. They resembled it to praying, and some considered caring for patients beyond praying. A male nurse with 27 years of experience said: “… I entered this job in order to serve and get close to God. So even if I’m bothered a lot, because I believe my job is like prayers, and not much material objective is behind it, tiredness makes no sense. On the contrary, it warms my heart.”

The same approach was stated by a male nurse with 16 years of experience: “… maybe the best justification of nursing despite all the trouble is that we work for the sake of God. Our aim is to serve the worshippers of God… so, at times of trouble, if you trust God, many problems will be solved easily and they don’t preoccupy you. You get along with yourself more easily…”

### 3.3 Spiritual Reward

Though having selected nursing as a profession to make money, the participants considered spiritual rewards in nursing important. They valued spiritual rewards so much that nursing problems became more bearable for them. A female nurse with 22 years of experience said: “… another issue is the spiritual aspect of the job. I mean, if you work for God, he pays you well. That we women with all these problems, and pressures of life and physical problems come to work is because of the goodwill prayers of patients. This is so precious for us. It’s a positive motivation factor for us…”

A male nurse, with all the heavy burden of life and its high expenses on his shoulders, disregards the material aspect and considers the patient as a dignified and invaluable being and says: “Frankly speaking, at the end of the month I’m surprised how I could make ends meet… I think without God’s blessings in my life, its patients’ goodwill prayers, and nothing else.”

### 3.4 Holiness of the Job

Spiritual outlook on the job comprised the majority of the attitude in the participants in that they preferred patients’ comfort to theirs. They considered caring a kind of praying. A female nurse with 8 years of clinical experience said: “In fact, for us nurses who follow Her Holiness Zeinab, being tired makes no sense. Because we believe that nursing is a kind of praying. For example, Ayatollah Tabatabaie said: I am ready to exchange the fruit of 70 years of my praying with one night of caring for patients. This shows the value of the job, and that the reward of our job can’t be measured in spiritual terms.”

A male nurse with 19 years of clinical experience said: “I believe nursing is a holy job because in Islam in addition to caring for patients, visiting them is stressed a lot. So when you have a spiritual approach to your job, all the problems that nurses in some other countries face are less in our country or places where there is a little religious attitude.”

### 3.5 Spiritual Journey

Further to considering care for patients a religious duty and choosing this profession because of being holy, the participants mentioned traveling to holy and religious places as a factor in reducing their fatigue. For example, a male nurse with 24 years of clinical experience said: “…with all my experience, I try to travel at least once a year, and a religious trip. When you pilgrim, all your tiredness is gone.”

Moreover, some nurses mentioned weekend and the role of having fun in reducing burnout as one of the strategies they use to adjust with burnout. In this regard, a male nurse with 23 years of clinical experience said: “Something I have vividly experience is traveling and having fun and the stuff. I mean a trip, a pilgrimage can be really effective. I don’t remember anything else or maybe I haven’t tried.”

## 4. Discussion

The present study aimed to study and determine the strategies used by nurses to adjust with professional burnout. The results of this study, based on objective experiences of the participants, showed that spirituality is one of the most important strategies to overcome nurses’ burnout. Most nurses considered provision of appropriate nursing services for patients based on their religious teachings. They believe what helps them to tolerate all the pressures and difficulties is trusting God and having a spiritual look at nursing.

Religion and religious beliefs, besides providing security and health for the community (Ganji & Hosseini, 2010) have been mentioned in Muslim’s holy book, Quran, as one of the stress reducing factors. Therefore, someone who believes Quran is his guide and involves it in all aspects of his life, sees all the trouble as a divine test decreed by God. He stands against the stressing factors hoping to resolve them, sees the future bright, and believes remembering God relaxes him (Ahmadi & Anoosheh, 2010).

In separate studies by Ravari (2012) and Emami (2007) on the role of spirituality on job satisfaction of nurses, it was revealed that spirituality was an important and inseparable part of nurses’ job satisfaction. Furthermore, it was a source of help for patients to adjust with disease process and other stressing life situations (Koenig, 2008).

The participants stated that nursing care was a religious duty and that they paid more attention to God’s satisfaction and its spiritual reward than its financial aspect. Therefore, they do not feel tired much because the objective is God’s satisfaction. Almost all studies on the concept of spirituality in nursing literature are related to key elements of self, others and God (Baldacchino & Draper, 2001). Therefore, on the basis of this attitude, nursing care for patients gains another meaning. Based on the experiences of the participant of the present study, it is concluded that they consider patient’s care empathy, friendship, brotherhood, religious insight, and finally a prayer. Omidvari (2008) believes spirituality is a personal experience that people gain through religious rituals. He mentions loving, helping others, and experiencing the joy that leads to life satisfaction as the common grounds for all spiritual manifestations.

Moreover, the participants mentioned passion and interest in job and their personal satisfaction as a moderating factor to overcome professional burnout. Other studies have also shown that religious coping is a source for emotional support, social support and hope. Therefore, those who use this coping strategy in their daily life suffer depression and anxiety less than others (Ganji & Hosseini, 2010; Yuen, 2008).

Koenig (2001) studied the relationship of religious beliefs and rituals with psychological health and social functioning. He found that religion affects psychological health by increasing the ability to cope with stress, creating a social support environment, creating hope and optimism to help create positive emotions toward better life, and being satisfied with life (Koenig, Larson, & Larson, 2001). He states in another study that people with stronger religious beliefs cope better with stressing situations, recover faster than non-religious people at time of sickness, experience fewer negative emotions, have less anxiety and enjoy a better social support. Therefore, spirituality and religious beliefs have a close relationship with mental health (Baldacchino & Draper, 2001; Culliford, 2002; Ganji & Hosseini, 2010; Greasley, Chiu, & Gartland, 2008; McNulty, Livneh, & Wilson, 2004), and are one of the factors affecting nurses’ coping with stresses caused at the time of caring for patients (Baldacchino & Draper, 2001; Cook et al., 2012).

Isikhan (2004) studied job stress and coping strategies of healthcare team of cancer patients, and concluded that the most common style used by healthcare team members was self-confidence style. In this study, the participants said relation with coworkers, loving the job, better financial status, fun and sports, spending more time with family, time management, planning, and attending scientific programs were among coping strategies for job stress. Nurses considered their peace of mind the fruit of patients’ goodwill prayer and the spiritual reward given to them by God. They wholeheartedly believed in holiness and the spiritual dimension of their job. Most of the participants mentioned the famous maxim that one night of nursing equals 70 years of prayers to show the holiness and spirituality of their job. Furthermore, Nurse’s Day in Islamic Republic of Iran is on the birthday of Her Holiness Zeinab, the great lady of Karbala, which shows the spiritual approach to this profession (Nikbakht Nasrabadi & Emami, 2006).

Nurses considered passion and interest in their job and its spirituality as important factors to help them tolerate the problems and stay in their jobs. They also said that although they did not have high salaries, they expected spiritual reward from God because of their beliefs. They experienced the effect of patients’ goodwill prayers in their life and believed the most important factor that relaxed them especially at night shifts was the smile and happiness of the patients and their families.

## 5. Conclusion

The results of this study emphasized that religious or spiritual beliefs give purpose and meaning to nursing interventions, help them tolerate the problems at work, and make nursing care pleasurable. They think of a patient as part of themselves. Therefore, although professional burnout is an important issue in nursing, attending to this dimension of their job is essential and healthcare authorities should pay a special attention to it. If nursing care is assessed in a quantitative and materialistic manner, the soul and body of the nurse will be worn out and his commitment will decrease.

**Ethical Considerations**

This research was approved by the Baqiyatallah University of Medical Sciences research committee. All participants were informed about the purpose of the study and gave a written consent for participation. They had the possibility to withdraw or interrupt their participation at any time.
